# A complete duplication of X chromosome resulting in a tricentric isochromosome originated by centromere repositioning

**DOI:** 10.1186/s13039-017-0323-7

**Published:** 2017-06-13

**Authors:** N. Villa, D. Conconi, D. Gambel Benussi, G. Tornese, F. Crosti, E. Sala, L. Dalprà, V. Pecile

**Affiliations:** 1 Medical Genetics Laboratory, Clinical Pathology Department, S. Gerardo Hospital, Monza, Italy; 2 School of Medicine and Surgery, University of Milano-Bicocca, Monza, Italy; 3 Medical Genetics, Institute for Maternal and Child Health I.R.C.C.S. “Burlo Garofolo”, Trieste, Italy; 4 Department of Pediatrics, Institute for Maternal and Child Health I.R.C.C.S. “Burlo Garofolo”, Trieste, Italy

**Keywords:** Chromosome complex rearrangement, Telomeric-centromeric X rearrangement

## Abstract

**Background:**

Neocentromeres are rare and considered chromosomal aberrations, because a non-centromeric region evolves in an active centromere by mutation. The literature reported several structural anomalies of X chromosome and they influence the female reproductive capacity or are associated to Turner syndrome in the presence of monosomy X cell line.

**Case presentation:**

We report a case of chromosome X complex rearrangement found in a prenatal diagnosis. The fetal karyotype showed a mosaicism with a 45,X cell line and a 46 chromosomes second line with a big marker, instead of a sex chromosome. The marker morphology and fluorescence in situ hybridization (FISH) characterization allowed us to identify a tricentric X chromosome constituted by two complete X chromosome fused at the p arms telomere and an active neocentromere in the middle, at the union of the two Xp arms, where usually are the telomeric regions. FISH also showed the presence of a paracentric inversion of both Xp arms.

Furthermore, fragility figures were found in 56% of metaphases from peripheral blood lymphocytes culture at birth: a shorter marker chromosome and an apparently acentric fragment frequently lost.

**Conclusions:**

At our knowledge, this is the first isochromosome of an entire non-acrocentric chromosome. The neocentromere is constituted by canonical sequences but localized in an unusual position and the original centromeres are inactivated.

We speculated that marker chromosome was the result of a double rearrangement: firstly, a paracentric inversion which involved the Xp arm, shifting a part of the centromere at the p end and subsequently a duplication of the entire X chromosome, which gave rise to an isochromosome. It is possible to suppose that the first event could be a result of a non-allelic homologous recombination mediated by inverted low-copy repeats.

As expected, our case shows a Turner phenotype with mild facial features and no major skeletal deformity, normal psychomotor development and a spontaneous development of puberty and menarche, although with irregular menses since the last follow-up.

## Background

The centromere structure plays different functions in the nucleus, all of these of great importance for the genome stability and cell division [1–3]. Its position in each chromosome is evolutionary-fixed, defines a p and q arms and represents a distinctive aspect of chromosome’s morphology, particularly useful in the karyotype reconstruction.

The centromere consists of α-satellite DNA sequences and other repeated sequences variable in number and type [4]. It is able to perform very complex protein binding in order to construct kinetochore, determining a fundamental role for a correct chromosome segregation. In the evolutionary scale it is species specific and highly divergent but, in spite of this, the overall architecture and composition of centromeric chromatin are similar among the different species. It appears that no specific sequences are essential to do an actively functional centromere: from several studies about centromeric inactivation and neocentromere formation, it is evident that the centromeric structure and function are the results of epigenetic events [5].

Neocentromeres, identified firstly by patients with clinical picture, are rare and considered chromosomal aberrations, because a non-centromeric region/locus evolves in an active centromere by mutation (gain of function) [6–8]. Few cases with an activation of a neocentromere without chromosomal rearrangements, together with an inactivation of canonical centromere (centromere repositioning) have been described [9].

The chromosome stability is defined by the presence of a number of TTAGGG repeats, called true telomeres, at the end of DNA molecules. Telomeric-like sequences were also described mapping the internal sites along the genome, but peculiarly at the peri- and centromeric regions of each chromosome [10].

## Case presentation

### Materials and methods

#### Chromosome analysis

Amniotic fluid was cultured using standard techniques and the chromosome analysis was based on in situ chromosome preparations obtained from independent cultures. Peripheral blood metaphases were obtained from phytohaemagglutinin-stimulated lymphocytes, cultured with Synchro kit (Celbio) according to manufacturer’s protocol.

Chromosome analysis was carried out applying QFQ banding according to routine procedures, and karyotypes were reconstructed following the guidelines of ISCN 2016 [11]. RBA banding were performed using standard protocols.

#### FISH analysis

Fluorescence in situ hybridization (FISH) was carried out as previously reported for the homemade probes [12] and according the manufacturer’s protocol for the commercial ones. To characterize the X anomalous, the following commercial probes were applied: centromeric X alpha-satellite (DXZ1, Xp11.1-q11.1), specific Xp telomere (DXYS129, Xp22.3) and common telomeric sequences (TTAGGG) (Oncor). Moreover BAC (Bacterial Artificial Chromosomes) probes were applied: RP11-167P23 (Xp11.22) and RP11-217H1 (Xq13.2–21.1) (Wellcome Trust Sanger Institute).

### Clinical report

A couple requested assisted reproductive technology because of 3 years of sterility. An intracytoplasmic sperm injection (ICSI) was performed and a twin pregnancy achieved. At 20th week of gestation, the morphological fetal examination at ultrasound evidenced one fetus with normal auxological parameters and the second one showing hyperechogenous focus and short limbs (femur length 27 mm, 19th percentile).

The spontaneous delivery occurred at 37 weeks of gestation. The first female baby was 2560 g in weight (10th - 25th percentile) and the second one was 2050 (<3rd percentile). At two months of life, the first twin was grown at 25th percentile whereas the second was at 3rd; after 8 months of life the first baby was at 75th percentile in height and 80th in weight, the second one was 10th in height and 25th in weight. The psychomotor development appear to be in the normal range for both the twins.

The girls are now 13-year-old. Echocardiographic study in the twin with lower growth ruled out cardiac anomalies. No kidney abnormalities were detected on ultrasound studies. She has mild facial features, with low nasal bridge and short nose, flat midface, low posterior hairline, pigmented nevi, but no Madelung deformity nor cubitus valgus. She suffered several episodes of recurrent infections of the middle ear only during infancy, without hearing loss.

Since the age of 3 and a half, she has been treated with somatropin for slowing of growth, now at the dose of 36 mcg/kg/day. Her height is now 149.5 cm (10-25th percentile), with a mid-parental height of 157.5 ± 8 cm cm (10-25th percentile) and a bone age of 14 years (according to Greulich & Pyle).

She developed spontaneous puberty at the age of 9 years and 8 months with spontaneous menarche at the age of 11 years and 4 months, with irregular menses hitherto (5 periods since menarche). Her pubertal status is now B4, Ph4, A3. Her body mass index (BMI) has been always in the upper percentiles, despite dietitian counseling; her BMI is now 24.21 kg/m^2^ (overweight, 85th percentile) with no laboratory evidence of diabetes mellitus and no hypertension.

Screening for Hashimoto’s thyroiditis and celiac disease have so far tested negative, although she carries DQ8 heterodimer (HLA-DQA1*03, HLA-DQB1*03:02), classically associated with celiac disease.

She had a normal psychomotor development and now is about to finish high school with good performance.

### Cytogenetic results

Amniocentesis was requested for the twin with hyperechogenous focus and short limbs in order to exclude the presence of Down Syndrome. Interphasic FISH on uncultured amniocytes with specific probe for Down Syndrome region showed a disomic pattern for chromosome 21 (data not shown).

**Fig. 1:**
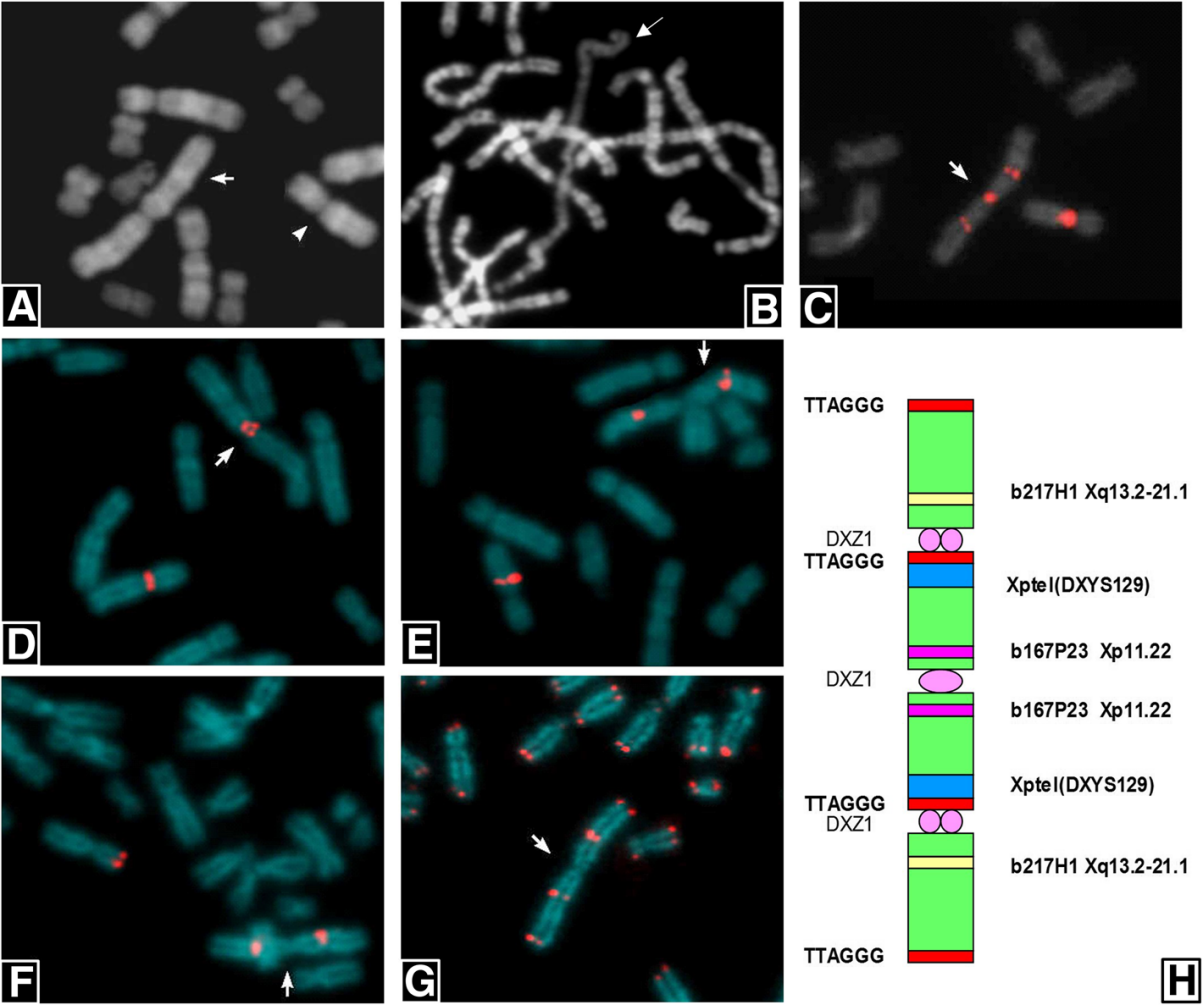
Cytogenetics and FISH characterization of marker. **a** QFQ banding of the normal chromosome X (*right*) and the marker (*left*). **b** RBA banding shows the inactivation of the marker (*arrow*) **c** FISH analysis with the specific probe for Xp11.1-q11.1 alpha-satellite (DXZ1, *red signals*) on the normal chromosome X and the marker (*arrow*). **d** FISH analysis with b167P23 BAC probe (Xp11.22, *red signals*) on the normal chromosome X and the marker (*arrow*). **e** FISH analysis with b217H1 BAC probe (Xq13.2–21.1, *red signals*) on the normal chromosome X and the marker (*arrow*). **f** FISH analysis with the specific probe for Xp/Yp telomere (DXYS129, *red signals*) on the normal chromosome X and the marker (*arrow*). **g** FISH analysis with the probe for common telomeric sequences (TTAGGG, *red signals*) on all chromosomes. The arrow indicates the marker. **h** Schematic representation of marker chromosome structure

Fetal karyotype analysis revealed a normal female (46,XX) for the first fetus and the presence of a mosaic (45,X/46,X,+mar) in the anomalous one. The X monosomy was observed in eight colonies and the presence of marker chromosome in other five different colonies deriving from four independent cultures. It was possible to recognize the marker morphology as a double apparently complete chromosome X, probably an isochromosome, sized more than chromosome 1 (Fig. 1a).

The unusual aspect was represented by the presence of an active centromere (AC, primary constriction) in the middle of the marker, at the union of the two Xp arms, where usually the telomeric regions are located. X inactivation study by means of RBA banding evidenced that the marker was inactive in all analysed cells (Fig. 1b).

In order to clarify the marker structure several FISHs were performed (Table 1).

**Table 1:** FISH summary

FISH probe	Chromosome region	Signals
DXZ1	Xp11.1-q11.1	+++
b217H1	Xq13.2–21.1	++
b167P23	Xp11.22	++ (Xp inversion)
DXYS129	Xp22.3	++
Common telomeres (TTAGGG)	Xptel, Xqtel	++++

The centromeric X alpha-satellite probe (DXZ1) showed an intense positive signal in the middle of the chromosome (new AC) and other two positive regions equally distant from the new centromere in the canonical position (Fig. 1c). These two centromeres were inactive (ICs) as indicated by the presence of two distinct signals, one for each chromatid.

Using BAC probes mapped in Xp11.22 (b167P23) and Xq13.2–21.1 (b217H1) an Xp paracentric inversion involving the entire p arm was identified (Fig. 1d, e, and h). In fact, the two hybridization signals for the 167P23 BAC probe were located at both sites of the AC instead near the ICs (Fig. 1d, h).

The specific Xp telomere probe (DXYS129) showed two hybridization signals localized near the two ICs (Fig. 1f) confirming the Xp inversion. The common telomeric sequences (TTAGGG) were evidenced in 4 different positions: 2 in correspondence of Xq telomeres and 2 next to the ICs (Fig. 1g, h).

The karyotype, defined following the International System of Chromosome Nomenclature 2016 [11], was: mos 45,X/46,X,der(X).ish inv(X)(p11.21p22.3)psu itrc(X)(p22.3)(DXZ1+++, b167P23++, b217H1++, DXYS129++, TTAGGG++++).

The peripheral lymphocytes at birth showed 46 metaphases with 45,X karyotype (23%) and 42 metaphases 46,X,psu itrc(X)(21%) out of total of 200 cells. Interestingly, the other 112 metaphases (56%) showed a shorter psu itrc(X) for the loss of an entire Xq arm. Only few metaphases maintain this fragment.

**Fig. 2:**
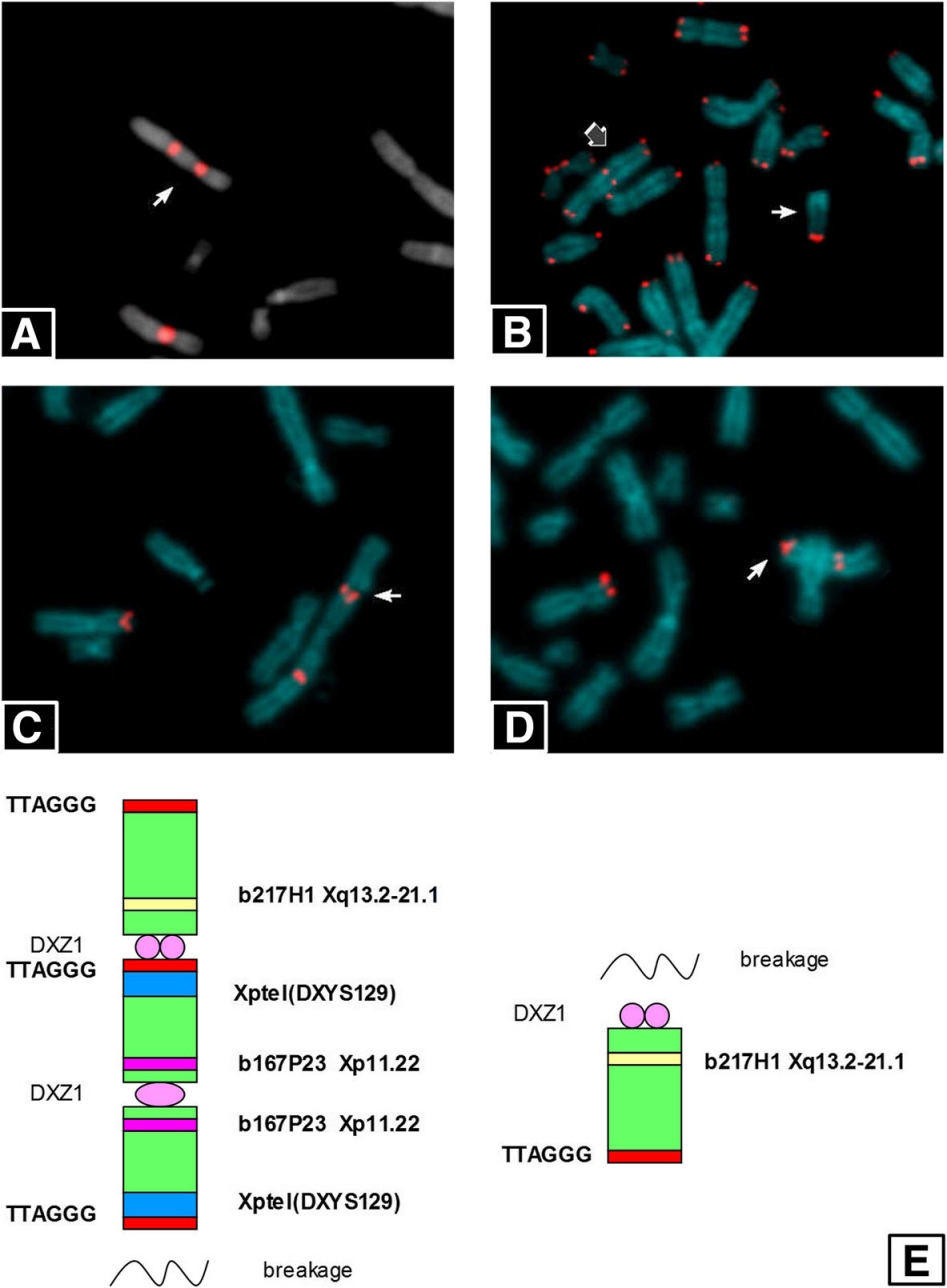
FISH characterization of deleted marker. **a** FISH analysis with the specific probe for Xp11.1-q11.1 alpha-satellite (DXZ1, *red signals*) on the normal chromosome X and the deleted marker (*arrow*). **b** FISH analysis with the probe for common telomeric sequences (TTAGGG, *red signals*) on all chromosomes. The *grey* and the *white arrows*, indicate the deleted marker and the acentric fragment respectively. **c** FISH analysis with the specific probe for Xp/Yp telomere (DXYS129, *red signals*) on the normal chromosome X and the entire marker. The arrow indicates the possible breaking point. **d** FISH analysis with the specific probe for Xp/Yp telomere (DXYS129, *red signals*) on the normal chromosome X and the deleted marker (*arrow*). **e** Schematic representation of deleted marker structure

The breakpoint was identified between the Xp TTAGGG sequences and the IC, as shown in Fig. 2a where the third centromeric signal of DXZ1 probe is not visible, probably retained by the lost fragment, while TTAGGG sequence was maintained on the deleted psu itrc(X) (Fig. 2b). An instability figure with the breaking point and the shorter psu itrc(X) were showed in Fig. 2c, d respectively after FISH with Xp specific telomere (DXYS129 probe). A schematic representation clarify the mechanism (Fig. 2e).

**Fig. 3:**
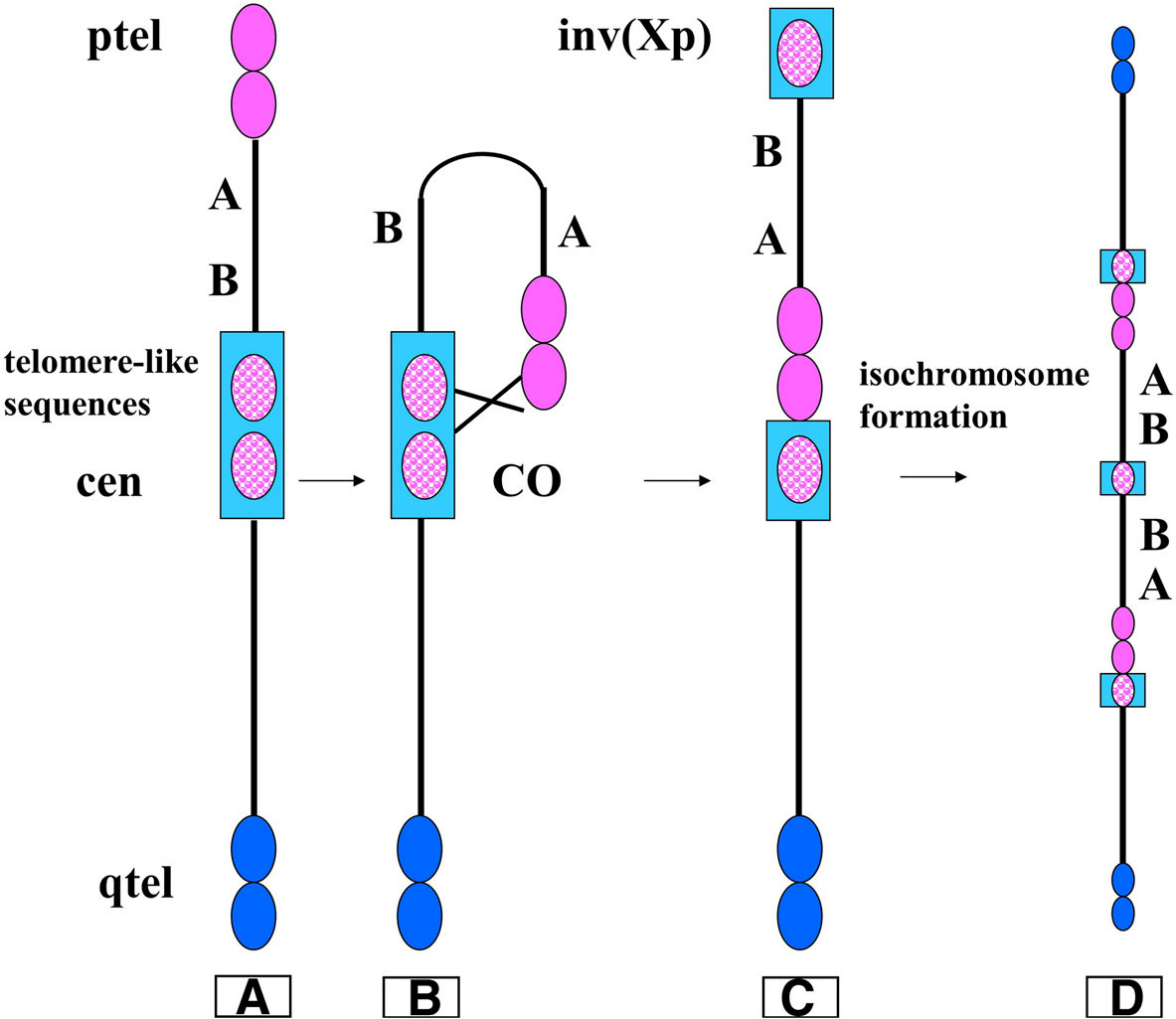
Hypothetical mechanism of marker formation. **a** Normal chromosome X. **b** Crossing-over mechanism between Xp telomere and the centromeric telomere-like sequences. **c** Paracentric inversion involving the Xp arm that results in a shift of the p end of centromere. **d** Duplication of the entire X chromosome, that gives rise to an isochromosome X

Summarizing, the FISH characterization revealed that the abnormal X chromosome was the result of a double rearrangement: firstly, a paracentric inversion which involved all the Xp arm (Fig. 3b), shifting a part of the centromere at the p end (Fig. 3c) and, subsequently, a duplication of the entire X chromosome, which gave rise to an isochromosome (Fig. 3d).

Parental karyotypes were normal and the Xp paracentric inversion was excluded using FISH (data not shown). The molecular karyotype (array-CGH) was not performed because the mosaic condition (monosomy X and trisomic X) can result in a final disomic situation for all X chromosome. A study by means of microsatellite polymorphisms mapped on X was performed (data not shown) and the X chromosomes resulted inherited from both parents, suggesting a meiotic II or postzygotic error. Finally, based on clinical request, the Y sequences were excluded (data not shown).

## Discussion and conclusion

Fetal karyotype on a twin pregnancy from ICSI showed a mosaic monosomy X with a second cell line with 46 chromosomes and a complex chromosome X rearrangement in the twin with short limbs and hyperecogenous focus. After molecular cytogenetic investigation the rearrangement was characterized as an isochromosome of entire X chromosome with new functional centromere with canonical alpha-satellite sequences in the middle of the chromosome.

Interestingly the isochromosome presented a not inherited paracentric inversion of both Xp arms. Instability figures on peripheral blood metaphases were observed and about 56% of cells showed a deleted psu itrc(X) chromosome because of the loss of a q arm.

At our knowledge, this is the first isochromosome of an entire non-acrocentric chromosome. Several aspects characterize this isochromosome X [i(X)]: first, the AC is a neocentromere because ectopic, but it is canonic for structure, on the other hand, the original centromeres were inactivated probably due to the rearrangement and the loss of sequences needed for the correct centromeric function.

In literature, Hemmat et al. [13] described a neocentric X-chromosome in a girl showing Turner-like syndrome, but the neocentromere was analphoid and no signals were detected after FISH with X-centromere specific probe or all human centromeres. The i(Xq) was constituted of two q arms and only two partial p arms differently from case here presented.

A tricentric X chromosome has also been described: it showed only one canonical active centromere distally positioned. The complex rearrangement produced firstly an i(Xq) dicentric followed by a second event of breakage resulting in a second i(Xq) with three centromeres. The detection was prenatally and the pregnancy terminated, showing minimal fetal anomalies at post-mortem examination [14].

The X genomic architecture is important to understand the apparently high frequency of rearrangement. It has been reported a fine study on X genomic organization with particular regard to Inverted Repeat (IR) structures. The authors described in the region Xp11.22 (where occurred the breakpoint of Xp paracentric inversion in the case here presented) the presence of 24 large IRs that represent the 25% of all human genomic IRs and show more than 99% of identity [15].

More recently, Dittwald P et al. [16] described a genome wide analysis of the Inverse Paralogous Low-Copy Repeats (IP-LCRs) that cause genome instability in every chromosome and are localized mainly in pericentromeric and telomeric regions. The authors concluded that IP-LCRs probably are loci of susceptibility for genomic instability by non-allelic homologous recombination (NAHR) that are involved in the aetiology of many genetic diseases.

Based on these data and looking at the case here described it is possible to suppose that the first event, inversion involving almost entire Xp arm, could be a result of a NAHR mediated by an IP-LCR that repositioned some of alpha-satellite repeats on telomere and viceversa (Fig. 3).

The interstitial telomeres can be hotspots of instability and could explain the fragility observed in this psu itrc(X) [10, 17]. However, it is impossible to understand if both the q arms of psu itrc(X) were fragile, due to perfect symmetry of this chromosome, but only one fragile site per metaphase was always present and never both simultaneously.

Finally, it is very suggestive the hypothesis regarding origin and evolution of centromere in eukaryotic chromosomes advanced by Villasante A et al., and integrated by Slijepcevic P et al. [18, 19]. They affirmed that centromeres were derived from telomeres and in the case here described both DNA types are involved resulting in this new X chromosome.

It is difficult to evaluate the parental origin of this rearrangement, even if a meiosis II or a postzygotic event could be suggested. In literature, structural abnormalities of the X chromosome appear equally likely to occur in either parent: the frequency of breaks is higher in paternal meiosis, but the probability of recombination errors between both X chromosomes is only maternal, giving rise to equal proportions [20].

The literature reported several structural anomalies of X chromosome and they influence the reproductive capacity of the woman or have a phenotypic effect. For example, X chromosome anomalies are estimated to constitute up to 13% of premature ovarian failure cases [21] and 25% of Turner syndrome cases.

Turner’s syndrome is characterized by the absence of all or part of a normal second X chromosome and occurs in one in 2500 female births [22]. It is generally associated to congenital lymphedema, short stature and gonadal dysgenesis, but physical manifestations differ according to chromosomal rearrangements. For example, the presence of a ring or marker chromosome confers an increased risk of mental retardation and atypical phenotypic features while the loss of the Xp results in a full phenotype [22].

Our case shows a Turner phenotype with mild facial features and no major skeletal deformity, no heart or kidney abnormalities, normal psychomotor development, overweight with no diabetes nor hypertension, no Hashimoto’s thyroiditis nor celiac disease since now. She also displayed growth slowing at the age of 3.5 years with a good response to somatropin treatment and near-final height within the mid*-*parental target range and a spontaneous development of puberty and menarche, although with irregular menses since the last follow-up.

## Abbreviations

AC: Active centromere
Array-CGH: Array comparative genomic hybridization
BAC: Bacterial artificial chromosomes
BMI: Body mass index
FISH: Fluorescence in situ hybridization
i(X): Isochromosome X
IC: Inactive centromere
ICSI: IntraCytoplasmic sperm injection
IP-LCRs: Inverse paralogous low-copy repeats
IR: Inverted repeat
NAHR: Non allelic homologous recombination
